# Retracted: Physiochemical and Phytochemical Properties of Wax Apple (*Syzygium samarangense* [Blume] Merrill & L. M. Perry var. Jambu Madu) as Affected by Growth Regulator Application

**DOI:** 10.1155/2021/9503057

**Published:** 2021-03-09

**Authors:** The Scientific World Journal

*The Scientific World Journal* has retracted the article titled “Physiochemical and Phytochemical Properties of Wax Apple (*Syzygium samarangense* [Blume] Merrill & L. M. Perry var. Jambu Madu) as Affected by Growth Regulator Application” [1] due to unreliable data and redundant publication.

The results show inconsistent overlaps with the authors' other articles published from 2011–15:

- Mohammad Moneruzzaman Khandaker, Abm Sharif Hossain, Normaniza Osman, and Amru Nasrulhaq Boyce, “Growth, yield and quality responses to gibberellic acid (GA3) of wax apple *Syzygium samarangense* var. Jambu air madu fruits grown under field conditions,” *African Journal of Biotechnology*, vol. 10, no. 56, pp. 11911–11918, September 26, 2011, https://doi.org/10.5897/AJB.9000278 [2].
- Mohammad Moneruzzaman Khandaker, Amru Nasrulhaq Boyce, Normaniza Osman, Faruq Golam, M. Motior Rahman and Sofian-Azirun, M., “Fruit development, pigmentation and biochemical properties of wax apple as affected by localized application of GA3 under field conditions,” *Brazilian Archives of Biology and Technology*, vol. 56, no. 1 Curitiba Jan./Feb. 2013, https://doi.org/10.1590/S1516-89132013000100002 [3].
- Mohammad Moneruzzaman Khandaker, Abm Sharif Hossain, Normaniza Osman, Nashriyah Mat, and Amru Nasrulhaq Boyce, “Growth, yield and postharvest quality of wax apple as affected by naphthalene acetic acid Application,” *Revista Brasileira de Fruticultura*, vol. 37, no. 2, pp. 410–422, 2018, https://doi.org/10.1590/0100-2945-062/14 [4].
- Mohammad Moneruzzaman Khandaker, Ali Majrashi, and Amru Nasrulhaq Boyce, “The influence of gibberellic acid on the chlorophyll fluorescence, protein content and PAL activity of wax apple (*Syzygium samarangense* var. jambu madu) fruits,” *Australian Journal of Crop Science*, vol. 9, no. 12, pp. 1221–1227, 2015, http://www.cropj.com/khandaker_9_12_2015_1221_1227.pdf [5].
- Mohammad Moneruzzaman Khandaker, Normania Osman, Abm Sharif Hossain, Golam Faruq, and Amru Nasrulhaq Boyce, “Effect of 2,4-D on Growth, Yield and Quality of Wax Apple (*Syzygium samarangense,* (Blume) Merrill & L. M. Perry cv. Jambu Madu), Fruits,” *Sains Malaysiana*, vol. 44, no. 10, pp. 1431–1439, 2015, http://www.ukm.my/jsm/pdf_files/SM-PDF-44-10-2015/08%20Mohammad%20Moneruzzaman.pdf [6].

Table 1 in [1] shows the same results as follows:

- Table 2 in [2] for fruit juice (mL/100 g) values and variance, except for the value for GA_3_ 100 (78 and 80, respectively)
- Table 1 in [3] for TSS (°Brix) values and variance, except for the value for GA_3_ 50 (11.5 vs. 10.5, respectively)
- Table 1 in [3] for titratable acidity (%) variances
- Table 3 in [4] for pH, expect for the value for control (4.90 and 4.92, respectively)
- Table 3 in [4] for TSS (°Brix) values for control and NAA 10
- Table 3 in [4] for TA values

Table 2 in [1] shows the same results as follows:

- Table 2 in [3] for total sugar (mg/100 g)

Table 3 in [1] shows the same results as follows:

- Table 2 in [2] for phenol mg GAE/100 g values and variances, except the value for GA_3_ 100 (552 vs. 752, respectively)
- Table 3 in [2] for chlorophyll (mg/L) values and variances
- Table 3 in [2] for carotenoid (*μ*g/g) values and variances, with the same variances but not the same values also appearing in Table 3 in [3]
- Table 2 in [3] for flavonoids (mg CE/100 g) values and variances, with the variances for the control and GA_3_ 20 also appearing in Table 2 in [2]
- Table 2 in [3] for anthocyanin values and variances (mg/100 g and mg/L, respectively), with the decimal places of the control and GA_3_ 20 values and variances and of the GA_3_ 50 and GA_3_ 100 values also being the same in Table 3 in [2]

Table 1 in [2] also shows the same results as follows:

- Table 1 in [3] for yield (kg) values and variances
- Table 1 in [3] for fruit drop (%) variances and GA_3_ 50 value
- Table 1 in [3] for average fruit weight (g) for variances
- Table 1 in [3] for fruit set (%) for control variance and GA_3_ 20 value and variance

Table 2 in [2] also shows the same results as follows:

- Table 1 in [3] for K+ content (mg/kg) variances

The studies [1, 5] report one season of overlapping data on GA_3_ treatment affecting chlorophyll and anthocyanin, i.e., December 2010–May 2011 in Banting.

The studies [1, 6] report experiments using 2,4-D during the same period (2008–11) in the same places (Klang and Banting). The only common outcome is total sugar, reported in [1] as mg/100 g and in [6] as g/100 g pulp, but the values are not the same. The corresponding author said that total fruit was used in [1] and edible fruit pulp in [6].

The corresponding author said that some data were reused due to premature fruit drop in some experiments, but the underlying data are no longer available.

## References

[1] Khandaker Mohammad Moneruzzaman, Boyce Amru Nasrulhaq, Osman Normaniza, Hossain Abm Sharif (2012). Physiochemical and Phytochemical Properties of Wax Apple (*Syzygium samarangense* [Blume] Merrill & L. M. Perry var. Jambu Madu) as Affected by Growth Regulator Application. *The Scientific World Journal*.

[2] Khandaker Mohammad Moneruzzaman, Hossain Abm Sharif, Osman Normaniza, Boyce Amru Nasrulhaq (2011). Growth, yield and quality responses to gibberellic acid (GA3) of wax apple *Syzygium samarangense* var. Jambu air madu fruits grown under field conditions. *African Journal of Biotechnology*.

[3] Khandaker Mohammad Moneruzzaman, Boyce Amru Nasrulhaq, Osman Normaniza, Golam Faruq, Rahman M. Motior, Sofian-Azirun M. (2013). Fruit development, pigmentation and biochemical properties of wax apple as affected by localized application of GA3 under field conditions. *Brazilian Archives of Biology and Technology*.

[4] Khandaker Mohammad Moneruzzaman, Hossain Abm Sharif, Osman Normaniza, Mat Nashriyah, Boyce Amru Nasrulhaq (2015). Growth, yield and postharvest quality of wax apple as affected by Naphthalene acetic acid application. *Revista Brasileira de Fruticultura*.

[5] Khandaker Mohammad Moneruzzaman, Majrashi Ali, Boyce Amru Nasrulhaq (2015). The influence of gibberellic acid on the chlorophyll fluorescence, protein content and PAL activity of wax apple (*Syzygium samarangense* var. Jambu madu) fruits. *Australian Journal of Crop Science*.

[6] Khandaker Mohammad Moneruzzaman, Osman Normania, Hossain Abm Sharif, Faruq Golam, Boyce Amru Nasrulhaq (2015). Effect of 2,4-D on growth, yield and quality of wax apple (*Syzygium samarangense*, (blume) Merrill & L. M. Perry cv. Jambu madu) fruits. *Sains Malaysiana*.

